# Visual disorders and mal de debarquement syndrome: a potential comorbidity questionnaire-based study

**DOI:** 10.2144/fsoa-2022-0022

**Published:** 2022-09-14

**Authors:** Cherylea J Browne, Paul Fahey, Stella R Sheeba, Margie H Sharpe, Mark Rosner, Debby Feinberg, Viviana Mucci

**Affiliations:** 1 School of Science, Western Sydney University, Sydney, NSW 2560, Australia; 2 Translational Neuroscience Facility (TNF), School of Medical Sciences, UNSW Sydney, NSW, 2033, Australia; 3 Brain Stimulation and Rehabilitation (BrainStAR) Lab, School of Health Sciences, Western Sydney University, Sydney, NSW, 2560, Australia; 4 School of Health Sciences, Western Sydney University, Sydney, NSW, 2560, Australia; 5 Dizziness & Balance Disorders Center, Adelaide, SA, 5000, Australia; 6 NeuroVisual Medicine Institute, Bloomfield Hills, MI 48302, USA

**Keywords:** balance disorder, binocular vision dysfunction, mal de debarquement syndrome (MdDS), mild traumatic brain injuries, vertical heterophoria, vestibular disorders, visual induced dizziness

## Abstract

**Aim:**

Mal de debarquement syndrome (MdDS) is a neurological condition characterized by a constant sensation of self-motion; onset may be motion-triggered (MT) or non-motion-triggered/spontaneous (NMT/SO). People with MdDS experience similar symptoms to those with vertical heterophoria, a subset of binocular visual dysfunction. Hence, we aimed to explore potential visual symptom overlaps.

**Methods:**

MdDS patients (n = 196) and controls (n = 197) completed a visual health questionnaire.

**Results:**

Compared with controls, the MdDS group demonstrated higher visual disorder scores and visual complaints. NMT/SO participants reported unique visual symptoms and a higher prevalence of mild traumatic brain injury.

**Conclusion:**

Our findings suggest visual disorders may coexist with MdDS, particularly the NMT/SO subtype. The difference in visual dysfunction frequency and medical histories between subtypes, warrants further investigation into differing pathophysiological mechanisms.

## Plain language summary

MdDS is a condition where patients feel like they are always on a boat. It is typically triggered by passive motion-events (cruises, flights, etc.), but can develop after non-motion events. People with MdDS can experience symptoms like those with certain visual disorders, therefore we wanted to see if there were overlaps between these conditions. This study surveyed people with MdDS and individuals from the general population about visual health and found that the MdDS group reported a higher frequency of visual dysfunction symptoms. Compared with motion-triggered patients, non-motion patients reported unique visual symptoms. This demonstrates that visual disorders may coexist in MdDS.

## Tweetable abstract

Mal de debarquement syndrome #MdDS is a rare condition where patients feel like they are always on a boat. Given the overlap of symptoms between MdDS and a common visual disorder – these researchers turned their focus toward visual comorbidities and found some eye-raising results!

Mal de debarquement syndrome (MdDS) is a rare neurological condition characterized by a perception of persistent self-motion, often described as ‘rocking’, ‘swaying’ and ‘bobbing’. The underlying pathophysiology remains unclear; however, one theory suggests MdDS is the result of a maladapted vestibular ocular reflex (VOR) [1], triggered by prolonged exposure to passive motion (e.g., being a passenger in a boat, train, car, etc.). This is the most common onset cause, termed motion-triggered (MT) MdDS, though 10–25% of patients develop identical symptoms in the absence of prolonged motion, which is denoted as non-motion triggered (NMT) or spontaneous/other (SO) MdDS (NMT/SO) [1–4]. In addition to the self-perception of motion (which is considered to be the primary symptom), MdDS patients of both subtypes report a range of associated symptoms such as abnormal postural movements, unsteadiness, heightened visual sensitivity, migraine headache, visual induced dizziness (VID) and brain fog [3–8]. Some of these symptoms overlap with certain visual disorders; in particular, a specific form of binocular vision dysfunction (BVD), vertical heterophoria (VH) [4,8–11]. Individuals may be born with this dysfunction, although, in most cases, it is caused by head injury, eye operations and age-related ocular changes [12–14]. There are multiple BVD subsets; however, VH is the most common cause of the symptoms (which are in line with MdDS), with a reported range of 7–52%, with best estimates at affecting ~20% of the general population [15]. VH is defined as a subtle vertical misalignment of the lines of sight of the two eyes (with one line of sight being above the other), which becomes apparent only when there is a disruption in the ability to align the eyes in such a manner that sensory fusion cannot be maintained [15]. Though these misalignments may be subtle, the visual system must continually compensate (both mechanically – involving the eye muscles; and sensory – involving central processing centers) to maintain a single visual percept. This may lead not only to eye strain, but also dizziness and headache, possibly affecting other interrelated sensory systems.

It is well known that the visual, vestibular and peripheral proprioceptive systems are vital in maintaining postural control [16–19]. Further, the visual system plays a crucial role in multisensory integration and has been considered as the most important of these systems [20–23]. Visual information and gaze signals influence three major reflex pathways: the VOR, the vestibulospinal tract (VST) and the reticulospinal tract (RST). The VOR reflex stabilizes gaze during rapid head movements. It has been proposed that this reflex plays a major role in the pathophysiology of MdDS [1, 24]. The VST innervates the anti-gravity muscles thereby maintaining equilibrium (postural control) and locomotion in conjunction with input from the RST [16–18,25]. The influence of visual abnormalities and vestibular dysfunction have been previously observed in people with BVD. It was found that these individuals were unable to use effectively and/or sustain binocular vision because of disjunctive eye movements, in which the visual axes move toward or away from each other leading to problems with visual fixations and impairment of the way ocular inputs project to vestibular centers [26]. Conversely, somatosensory (proprioception) inputs influence the mobilization of the eyes and visual perception of surrounding space [27–30]. Therefore, visual dysfunctions or visual disorders such as VH (given its symptom similarities) may compromise the VOR and be a predisposing factor for the development of MdDS, especially if an underlying visual dysfunction causes dysfunctional integration of VOR suppression during passive motion, hence inducing MT MdDS.

Based on this premise, the major aims of the current study were twofold: firstly, to compare the frequency of visual disorders, visual dysfunction and mild head injury, general visual health data, eye operations and headache experience between people with MdDS and the general population; secondly, to compare the two subtypes of MdDS (MT and NMT/SO) to evaluate similarities and/or differences that could help categorize these two groups further. We hypothesized: a higher frequency of visual disorders and visual dysfunction in people with MdDS compared with the general population; and headache experience and medical histories (mild head injuries and eye operations) would reveal distinguishing features between people with MdDS and the general population, and also between the two MdDS subtypes. The findings of this study will advance our understanding of the MdDS population and highlight different patient characteristics in the two subtypes, ultimately enhancing diagnostic assessments, treatments and management strategies for people with MdDS.

## Materials and Methods

### Study population and recruitment

A cross-sectional study design was used. MdDS patients and healthy control subjects (general population) were recruited globally, and each respondent provided informed consent. Information about participating in the study was posted on the main MdDS support groups on Facebook, and the websites for MdDS Australia and the Dizziness and Balance Disorders Centre in Adelaide. The inclusion criteria [4] for MdDS participants was: ≥18 years old, patients reporting sensations of self-motion (rocking, swaying and bobbing) for longer than 1 month, where the symptoms could not be explained by another diagnosis, patients reporting MdDS symptoms after the exposure to passive motion (were allocated to the motion-triggered [MT] group), patients reporting the initial symptoms after a strong emotional or stressful event (e.g., childbirth, physical or emotional trauma, surgery, etc.) (were allocated to the non-motion triggered [NMT] group), or patients reporting similar symptoms without a clear motion event or any obvious cause (were allocated to the spontaneous/other [SO] group) (combined = NMT/SO group). To gather general population participants, the survey was shared by the authors to their existing institutional networks, and MdDS patients were encouraged to share the study information with their friends and family.

### Questionnaires

Data were self-reported. The questionnaires were distributed online, using Qualtrics XM (Qualtrics International Inc.). The questionnaire consisted of 49 items (Appendix – A.1), however only results relating to demographic details, associated MdDS symptoms, visual disorders and visual health, pre-existing medical conditions, head injuries, headache areas and VID are presented in this manuscript. Specific questionnaires were embedded within the customized questions created specifically for this study. The specific questionnaires administered were: the VH questionnaire (BVDQ) [15], a validated VH symptom assessment instrument which evaluates the potential VH symptom frequency [15]; and the Visual Vertigo Analogue Scale (VVAS) [31], which is used to determine the effects of visual stimulation on the provocation of dizziness. The VVAS evaluates patient symptoms in response to visual stimuli [32]; and the Situational Vertigo Questionnaire (SVQ), which provides a normalized score of the severity of symptoms provoked by disorienting environments, especially those patients with visual–vestibular conflict [33]. In addition, although the BVDQ gives an overall score, the questionnaire is multifaceted and covers seven symptom domains (i.e., 1. Headache, 2. Head tilt, 3. Vestibular, 4. Anxiety, 5. Binocular vision, 6. Reading, and 7. Standard vision). As the focus of this paper is on visual disorders and the vestibular system, all symptom domains except reading and anxiety, were considered in this manuscript. In response to each question, the respondent has the option of selecting: ‘Never’, ‘Occasionally (i.e., less than one-time per week)’, ‘Frequently (i.e., at least one-time per week)’ and ‘Always (everyday)’. To prevent non-responses, every question required a response before the respondent could proceed.

### Statistical analysis

Given the opt-in nature of participation, no formal sample size calculations were undertaken prior to data collection. If we assume that the respondents have the same characteristics as random samples and conduct a two-sided hypotheses test at the 0.05 significance level, the achieved sample size (196 – MdDS and 197 – general population) would provide 80% power to detect a difference between population means of 0.28 standard errors (Cohen's d = 0.28) as statistically significant, which is commonly regarded as a small effect. For categorical outcome variables, the observed sample size would have 80% power to detect a difference between 50 and 36% (or between 10 and 3%) as statistically significant at the 5% significance level. Statistical analysis was performed with SPSS version 25 (IBM Corp.). Sample data were summarized using means and standard deviations for numeric variables and percentages for the categorical variables. The distributions of all numeric variables were checked for symmetry, visually and using skewness statistics. Tabulations of categorical variables were reviewed for small categories. Differences between populations (MdDS vs general population and MT vs NMT/SO) were investigated using independent samples *t*-tests for numeric outcomes, and Pearson's χ^2^ or Fisher's exact test for categorical variables [34]. Linear, multinomial logistic, ordinal or negative binomial regression models were used for numeric, nominal, ordinal and count variables, respectively. Statistical significance was defined as p < 0.05 with p < 0.10 characterized as weak evidence at this exploratory stage. Results are not corrected for multiple testing, minimizing the risk of false negative results at this exploratory stage. Positive findings are presented for further investigation and confirmation.

## Results

People with MdDS diagnosed by specialists or self-diagnosed (believed they suffered from MdDS and were unable to get an official diagnosis) were recruited. Self-diagnosed patients were included in this study due to the lack of awareness of this condition among medical practitioners. Consequently, low awareness of this condition within the medical community means that some patients struggle to find a medical professional able to provide them with the correct diagnosis. As in previous studies, symptom description and patient history were evaluated carefully and when patients did not fit the criteria established by the guidelines [3,4] for MdDS, they were excluded from the study. No patients were excluded (in our study) since all ‘self-diagnosed’ respondents met the inclusion criteria summarized in ‘Study population and recruitment’.

### Epidemiology – sample description

All data pertaining to this section is in Table 1.

**Table 1.** Sample description

	MT MdDS	NMT/SO MdDS		MdDS (combined)	General population	
Respondent type, n (%)	146 (74.5%)	50 (25.5%)		196 (50.1%)	197 (49.1%)	
Age (years), mean (SD)	51.4 (11.9)	47.1 (14.9)	*t*_71.7_ = 1.853, p *=* 0.068^†^	50.3 (12.8)	50.5 (13.0)	*t*_391_ = -0.210, p = 0.834^†^
Gender, n (%)						
Female	136 (93.2%)	41 (82.0%)	**p = 0.028** ^‡^	177 (90.3%)	171 (86.8%)	p = 0.342 ^‡^
Male	10 (6.8%)	9 (18.0%)		19 (9.7%)	26 (13.2%)	
Age at onset (years), mean (SD)	44.1 (12.3)	38.3 (14.6)	*t*_194_ = 2.775, **p = 0.006**^†^	42.6 (13.2)	–	

† Independent Samples *t*-test (two-sided significance).

‡ Fisher's exact test (two-sided significance).

Detailed data of the general population group and MdDS patients; MT and NMT/SO, including group number, gender and age. Data for onset age for MdDS patients only.

Bold p values are statistically significant.

MdDS: Mal de debarquement syndrome; MT: Motion-triggered; NMT/SO: Non-motion triggered/spontaneous-other; SD: Standard deviation.

#### Respondent type

Three hundred and ninety-three responses were collected over two separate time periods which totaled 3 months. There was an equal distribution of respondents from the general population and from the MdDS population. The majority of MdDS respondents were of the MT subtype and a quarter were of the NMT/SO subtype. No patients were excluded (in our study) since all ‘self-diagnosed’ respondents met the inclusion criteria summarized in Study population and recruitment [3,4].

#### Gender & age

A higher proportion of females participated in the study, with a female to male ratio of approximately 9:1. When comparing the gender proportions between the MT and NMT/SO groups, there was a statistically significant higher proportion of males in the NMT/SO group than in the MT group, which had a female to male ratio of 8:2. Among those with MdDS, there was weak evidence that NMT/SO tended to be younger on average.

### MdDS-specific questions

All data pertaining to this section is presented in Table 1, apart from ‘Onset cause’ and ‘Associated symptoms’.

#### Onset cause

The most common cause of onset in the 146 MT respondents was a ‘boat/cruise’ (69.2%) followed equally by ‘flight’ and a ‘combination of vehicles’ (13.0% each). A smaller percentage of MT respondents reported ‘car’ (3.4%) and ‘train/metro’ (1.4%) as their cause of onset. Of the 50 NMT/SO respondents, 68.0% did not attribute any event for the onset of their symptoms. An injury/accident was reported in 6% of the group. NMT/SO also reported the following as onsets: stress (4%), an infection (4%), a vertiginous event episode (4%), using gym equipment (4%), childbirth (2%), surgery (2%), drug toxicity (2%), medication withdrawal (2%) and swimming (2%).

#### Age at onset

The average age of MdDS onset for both subtypes combined was 42.6 years (SD = 13.2). The MT group had a significantly higher average age of onset at 44.1 years (SD = 12.3), compared with 38.3 years (SD = 14.6) for the NMT/SO group.

#### Associated symptoms

Brain fog and unsteadiness were the most common associated symptoms for both MT and NMT/SO MdDS respondents. The NMT/SO subtype had a significantly higher proportion of respondents reporting double vision and tended to report nausea and ‘shaking’ peripheral vision more than the MT subtype, although the statistical evidence was marginal (Table 2).

**Table 2.** Associated symptoms

	MT (n = 146) (within group %)	NMT/SO (n = 50) (within group %)	Fisher's exact test (two-sided significance)	Total MdDS respondents, n = 196 (%)
Unsteadiness	117 (80.1%)	44 (88.0%)	0.285	161 (82.1%)
Brain fog	119 (81.5%)	37 (74.0%)	0.309	156 (79.6%)
Light sensitivity	104 (71.2%)	30 (60.0%)	0.160	134 (68.4%)
Anxiety	92 (63.0%)	34 (68.0%)	0.609	126 (64.3%)
Dizziness	56 (38.4%)	25 (50.0%)	0.183	81 (41.3%)
Depression	56 (38.4%)	24 (48.0%)	0.247	80 (40.8%)
Migraine	57 (39.0%)	21 (42.0%)	0.740	78 (39.8%)
Nausea	39 (26.7%)	21 (42.0%)	0.051	60 (30.6%)
‘Shaking’ peripheral vision	31 (21.2%)	17 (34.0%)	0.086	48 (24.5%)
Double vision	15 (10.3%)	17 (34.0%)	**<0.001**	32 (16.3%)

Comparison of the rate of associated symptoms expressed as a percentage of the MT and NMT/SO groups, and total percentage of all MdDS respondents.

Bold p values are statistically significant.

MdDS: Mal de debarquement syndrome; MT: Motion-triggered; NMT/SO: Non-motion triggered/spontaneous-other; SD: Standard deviation.

### Visual disorders and visual correction in the general population and people with MdDS

#### The presence of visual disorders

The majority of previously diagnosed visual disorders listed in the questionnaire were represented in equal proportions between the MdDS and general population groups (Table 3), with the exception of VID and BVD, where the MdDS group had significantly higher reports of official diagnoses than the general population group (p = 0.003 and 0.023, respectively). There was no significant difference in the frequency of these visual disorders between the two MdDS subtypes (Table 3). Please note, this does not consider those individuals who were not aware of having either of these conditions. However, the VVAS and SVQ questionnaires provides a quantitative evaluation scale of VID and identifies the presence of visual–vestibular conflict. The scores for both questionnaires were significantly higher in the MdDS group compared with the general population group (both p < 0.001). Within the MdDS group, there were no significant differences between the subtypes on the VVAS and the SVQ scores (Table 4). The mean BVDQ score was significantly higher in the MdDS group compared with the general population (p < 0.001). Additionally, the NMT/SO group had a higher BVDQ mean score compared with the MT group (p = 0.044) (Table 4).

**Table 3.** Pre-existing visual disorders

	MdDS relative to general population OR (95% CI)	p-value	NMT/SO relative to MT OR (95% CI)	p-value
Visual Induced Dizziness	22.3 (2.9,167.7)	**0.003**	2.1 (0.8,5.6)	0.123
Binocular visual dysfunction	5.8 (1.3,26.5)	**0.023**	2.6 (0.8,8.9)	0.130
Myopia	0.8 (0.6,1.2)	0.385	1.1 (0.6,2.2)	0.719
Hyperopia	0.8 (0.5,1.3)	0.351	1.1 (0.5,2.4)	0.828
Glaucoma	Too few		Too few	
Cataracts	0.9 (0.4,2.1)	0.847	2.2 (0.7,7.3)	0.195
Presbyopia	1.7 (0.9,3.2)	0.136	0.7 (0.2,2.0)	0.500
Astigmatism	0.6 (0.2,1.7)	0.317	1.5 (0.3,8.3)	0.657
None	1.1 (0.7,1.6)	0.805	1.1 (0.6,2.1)	0.770

Comparison of the frequency of previously diagnosed visual disorders in the general population group compared with the MdDS patients, and between MT and NMT/SO MdDS subtypes using logistic regression.

Bold p values are statistically significant.

CI: Confidence interval; MdDS: Mal de debarquement syndrome; MT: Motion-triggered; NMT/SO: Non-motion triggered/spontaneous-other; OR: Odds ratio; SD: Standard deviation.

**Table 4.** Visual Vertigo Analogue Scale, Situational Vertigo Questionnaire scores and Binocular Visual Dysfunction Questionnaire scores

				MdDS		
	General population	MdDS	p-value	MT	NMT/SO	p*-*value
VVAS score (SD) (out of 10)	0.7 (1.3)	4.5 (2.7)	*t_282.762_* = 18.041, **p < 0.001**	4.3 (2.6)	4.9 (2.8)	*t*_194_ = -1.318, p = 0.189
SVQ score (SD) (out of 4)	0.4 (0.6)	1.6 (1.0)	*t_308.623_* = 15.109, **p < 0.001**	1.6 (1.0)	1.8 (1.1)	*t*_194_ = -1.631, p = 0.105
BVDQ score (SD) (out of 75)	11.8 (10.3)	32.0 (14.9)	*t*_345.420_ = 15.627, **p < 0.001**	30.7 (14.7)	35.6 (15.1)	*t*_194_ = -2.026, **p = 0.044**

This table reports a summary of statistical and comparisons for the VVAS, SVQ scores and BVDQ scores of the general population group and MdDS patients, and between the MT and NMT/SO MdDS groups, using independent samples *t*-tests.

Bold p values are statistically significant.

BVDQ: Binocular Visual Dysfunction Questionnaire; MdDS: Mal de debarquement syndrome; MT: Motion-triggered; NMT/SO: Non-motion triggered/spontaneous-other; SD: Standard deviation; SVQ: Situational vertigo questionnaire; VVAS: Visual vertigo analogue scale.

#### Visual correction

Most individuals with correctable visual dysfunctions (i.e., myopia and hyperopia) indicated the use of prescription lenses. There were no significant differences between all groups on the use of prescription glasses/contact lenses. 76.6% of the general population group reported prescription glasses/contact use. 74.0% of MdDS respondents (p = 0.560), 75.3% of MT respondents and 70.0% of NMT/SO respondents (p = 0.460) used prescription glasses/contact lenses.

### BVDQ symptom domains

In addition to the overall BVDQ score, here we present the data for the relevant symptom domains (Table 5).

**Table 5.** Relevant Binocular Visual Dysfunction Questionnaire symptom domain questions

		Occasionally		Frequently		Always		p-value	Occasionally		Frequently		Always		p-value
		Control	MdDS	Control	MdDS	Control	MdDS		MT	NMT/SO	MT	NMT/SO	MT	NMT/SO	
Headache	Do you have headaches and / or facial pain?	81.6%	38.5%	18.4%	42.3%	0.0%	19.2%	**p < 0.001**	41.6%	30.2%	40.7%	46.5%	17.7%	23.3%	p = 0.391
	Do you have pain in your eyes with eye movement?	88.4%	62.0%	11.6%	22.8%	0.0%	15.2%	**p = 0.002**	67.9%	50.0%	18.9%	30.8%	13.2%	19.2%	p = 0.277
Head tilt	Do you experience neck or shoulder discomfort?	57.8%	22.7%	25.9%	36.4%	16.3%	40.9%	**p < 0.001**	27.7%	8.7%	36.2%	37.0%	36.2%	54.3%	**p = 0.013**
	Do you ever find yourself with your head tilted to one side?	41.7%	45.2%	28.6%	28.2%	8.3%	26.6%	**p < 0.001**	48.3%	37.8%	28.7%	27.0%	23.0%	35.1%	p = 0.362
Vestibular	Do you have dizziness and / or light headedness?	86.4%	25.9%	10.9%	38.5%	2.7%	35.6%	**p < 0.001**	27.3%	21.7%	42.2%	28.3%	30.5%	50.0%	p = 0.065
	Do you experience dizziness, light headedness, or nausea while performing closeup activities (computer work, reading, writing, etc.)?	81.3%	37.9%	6.3%	26.2%	12.5%	35.9%	**p < 0.001**	42.5%	25.6%	28.3%	20.5%	29.2%	53.8%	**p = 0.029**
	Do you experience dizziness, light headedness, or nausea while performing far-distance activities (driving, television, movies, etc.)?	79.2%	56.5%	12.5%	26.9%	8.3%	16.7%	p = 0.147	61.8%	43.8%	28.9%	21.9%	9.2%	34.4%	**p = 0.009**
	Do you experience dizziness, light headedness, or nausea when bending down and standing back up, or when getting up quickly from a seated position?	76.3%	31.5%	20.3%	32.7%	3.4%	35.8%	**p < 0.001**	32.5%	28.6%	35.0%	26.2%	32.5%	45.2%	p = 0.343
	Do you feel unsteady with walking, or drift to one side while walking?	74.0%	24.0%	20.0%	29.6%	6.0%	46.4%	**p < 0.001**	26.3%	17.4%	27.8%	34.8%	45.9%	47.8%	p = 0.423
	Does riding in a car make you feel dizzy or uncomfortable?	83.0%	53.4%	11.3%	20.7%	5.7%	25.%	**p = 0.002**	59.0%	42.1%	17.9%	26.3%	23.1%	31.6%	p = 0.527
Binocular vision	Do you experience poor depth perception or have difficulty estimating distances accurately?	71.0%	32.5%	16.1%	33.3%	12.9%	34.2%	**p < 0.001**	33.7%	28.6%	30.2%	42.9%	36.0%	28.6%	p = 0.484
	Do you experience double / overlapping / shadowed vision at far distances?	76.7%	45.0%	6.7%	30.0%	16.7%	25.0%	**p = 0.009**	39.0%	57.9%	39.0%	10.5%	22.0%	31.6%	p = 0.074
	Do you experience double / overlapping / shadowed vision at near distances?	72.7%	43.5%	18.2%	31.9%	9.1%	24.6%	**p = 0.021**	45.8%	38.1%	31.3%	33.3%	22.9%	28.6%	p = 0.844
	Do you experience glare or have sensitivity to bright lights?	63.1%	22.5%	23.1%	25.4%	13.8%	52.1%	**p < 0.001**	22.5%	22.5%	24.8%	27.5%	52.7%	50.0%	p = 0.970
	Do you close or cover one eye with near or far tasks?	62.2%	37.7%	27.0%	37.7%	10.8%	24.6%	p = 0.057	34.9%	44.4%	39.5%	33.3%	25.6%	22.2%	p = 0.824
Standard vision	Do you tire easily with close-up tasks (computer work, reading, writing)?	65.6%	32.1%	26.7%	27.0%	7.8%	40.9**%**	**p < 0.001**	33.6%	27.5%	27.7%	25.0%	38.7%	47.5%	p = 0.624
	Do you experience blurred vision with far-distance activities (driving, television, movies, chalkboard at school, etc.)?	64.4%	51.4%	20.5%	26.6%	15.1%	22.0%	p = 0.225	55.6%	39.3%	22.2%	39.3%	22.2%	21.4%	p = 0.192
	Do you experience blurred vision with close-up activities (computer work, reading, writing, etc.)?	54.1%	43.0%	33.8%	29.8%	12.2%	27.3%	**p = 0.039**	48.9%	27.3%	25.0%	42.4%	26.1%	30.3%	p = 0.076
	Do you blink to “clear up” distant objects after working at a desk or working with closeup activities (computer work, reading, writing, etc.)?	69.0%	39.2%	22.4%	33.3%	8.6%	27.5%	**p < 0.001**	40.9%	34.4%	30.7%	40.6%	28.4%	25.0%	p = 0.655

This table shows the scores on the BVDQ section between the general population and the MdDS group, between the MT and NMT/SO MdDS groups, using the Fisher's exact test.

Bold p values are statistically significant.

BVDQ: Binocular Visual Dysfunction Questionnaire; MdDS: Mal de debarquement syndrome; MT: Motion-triggered; NMT/SO: Non-motion triggered/spontaneous-other.

#### Headache

In the two headache domain questions, a significantly higher proportion of MdDS respondents indicated they ‘Always (everyday)’ ‘have headaches and/or facial pain’ (p < 0.001) and ‘pain in [their] eyes with eye movement’ (p = 0.002), compared with the general population. There were no significant differences between the MdDS subtypes for either of these domain questions (Table 5).

In addition, in the two head tilt domain questions a significantly higher proportion of MdDS respondents indicated that they ‘Always (everyday)’ ‘experience neck or shoulder discomfort*’* (p < 0.001) and ‘*f*ind [themselves] with [their] head titled to one side*’* (p <0.001) when compared with the general population. When comparing the two MdDS subtypes, the NMT/SO group had a higher proportion of ‘Always (everyday)’ ‘experiencing neck or shoulder discomfort*’* (p = 0.013) (Table 5).

#### Vestibular

Of the six vestibular domain questions, the MdDS group had a higher proportion of ‘Always (Everyday)’ in five of the six questions when compared with the general population (p values range from p < 0.001 and p = 0.002) (see Table 5 for full questions). The NMT/SO had a higher proportion of ‘Always (Everyday)’ when compared with the MT group for two vestibular domain questions: *‘*Do you experience dizziness, light headedness, or nausea while performing closeup activities?*’* (p = 0.029), and *‘*Do you experience dizziness, light headedness, or nausea while performing far-distance activities (driving, television, movies, etc.)?*’* (p = 0.009).

#### Binocular vision

Of the five binocular vision domain questions, the MdDS group had a higher proportion of ‘Always (Everyday)’ in four of the questions when compared with the general population (p values range from p < 0.001 and p = 0.021), with the fifth question at (p = 0.057) (see Table 5 for full questions). No differences were observed between the MdDS groups.

#### Standard vision

Of the four standard vision domain questions, the MdDS group had a higher proportion of ‘Always (Everyday)’ in three of the four questions when compared with the general population (p values range from p < 0.001 and p = 0.039) (see Table 5 for full questions). No differences were observed between the MdDS groups.

### Patient history

#### Previous eye operations

There were no significant differences in the proportion of respondents that had eye operations between the general population and the MdDS groups. There was weak evidence of a higher rate of eye operations in the MT group compared with the NMT/SO group (p = 0.078) (Table 6).

**Table 6.** Patient history

				MdDS		
	General population	MdDS	p*-*value	MT	NMT/SO	p*-*value
Eye operations	25 (12.7%)	33 (16.8%)	0.259	29 (19.9%)	4 (8.0%)	0.078
Mild traumatic brain injury/concussion	18 (9.1%)	23 (11.7%)	0.415	11 (7.5%)	12 (24.0%)	**0.004**

This table represents prior injuries and eye operations reported on the BVDQ in the general population compared with the MdDS groups, and between the MT and NMT/SO MdDS groups, using Fisher's exact test.

Bold p values are statistically significant.

BVDQ: Binocular Visual Dysfunction Questionnaire; MdDS: Mal de debarquement syndrome; MT: Motion-triggered; NMT/SO: Non-motion triggered/spontaneous-other.

#### Mild traumatic brain injury (concussion)

A Fisher's exact test indicated there were no significant differences in the rate of mild traumatic brain injuries (mTBI [concussion]) (p = 0.415) between the MdDS group and the general population group, although the NMT/SO group had a significantly higher rate of mTBI/concussion compared with the MT group (p = 0.004) (Table 6).

## Discussion

This study is the first to investigate the presence of visual disorders and visual dysfunction, and explore general visual health data, histories of mild head injury, eye operations and headache experience, in a group of MdDS sufferers compared with individuals from the general population, using customized and validated questionnaires. From the literature there is a clear crossover of symptoms between MdDS and VH, such as heightened visual sensitivity, migraine, headache, VID and brain fog, which is common in those with increased visual dependence [4,9,35]. The crossover of symptom profiles between these conditions is intriguing, given that the two are seemingly vastly different disorders. It is known that sensory reweighting toward the visual system is common in vestibular disorders, to gain spatial orientation and therefore maintain balance when vestibular inputs are impaired [33,36,37], however it may be possible, and we hypothesize, that people with MdDS had pre-existing visual deficits which rendered them more susceptible to developing the disorder.

### Epidemiology

Three hundred and ninety-three respondents completed the questionnaire, with even distributions between general and MdDS populations. Although there was no control for age and gender matching between the general population group and MdDS respondents; the average age matched between the groups and the gender proportions matched organically with a 9:1 female to male ratio. Respondents of the MT subtype represented three quarters of the surveyed MdDS population, and the remaining quarter were the NMT/SO subtype. The average age of the general and MdDS population groups was approximately 50 years, in line with previous studies [4]. The majority of the MdDS respondents were female, which concurs with findings of other studies reporting a prevalence of females in the MdDS population [3,38,39]. Interestingly, there was a higher proportion of male respondents of the NMT/SO group compared with the MT subtype. Despite being preliminary, this finding adds to the theory that these two MdDS subtypes may have different underlying pathologies. The MT susceptibility to developing MdDS may be directly related to female sex hormones [40,41] as it has been shown in previous work that the NMT/SO and the MT MdDS subtypes have distinct differences related to female sex hormones, i.e. the age of onset (commonly pre-menopausal age for NMT/SO and perimenopausal/menopausal for MT), hormonal aggravation of symptoms in NMT/SO patients, and a higher rate of polycystic ovarian syndrome in MdDS patients compared with the general population [3,4].

### MdDS respondents

#### Onset cause

Most of the MT subtype respondents developed symptoms after a boat trip or cruise, which has been previously shown to be the typical and most common onset for MdDS [4,42]. Some attributed the onset of their symptoms to traveling by plane or traveling on a combination of vehicles. A small percentage of MT respondents reported that their symptoms developed after traveling by car, train or metro. These results support findings of previous studies of MdDS onset and the low frequency oscillations commonly experienced on these types of motion experiences is hypothesized to disrupt the VOR and, consequently, the patient's velocity storage in the brainstem and cerebellum [3,4,6,41–44].

On the other hand, most of the respondents in the NMT/SO subtype were unable to attribute the onset of their symptoms to prolonged passive motion and reported that no precise trigger caused the onset of their symptoms. There were other respondents in this group who reported non-motion causes, which were diverse. Around 6% of the respondents in the NMT/SO group attributed their onset to an injury or an accident while some reported the onset of symptoms was caused by high levels of stress, an acute or chronic vestibular episode, drug toxicity, surgery, an infection, swimming, medication withdrawal, childbirth and exercising using gym equipment (e.g., stationary bike, treadmill). Disembarkation was not the cause of onset for the NMT/SO respondents. Therefore, this group does not meet the criterion for MT MdDS. As research progresses in this area, differences between MT and NMT/SO are emerging. This raises the issue that the NMT/SO subtype of MdDS should perhaps be re-classified as a subset of persistent postural-perceptual dizziness (PPPD) and not MdDS [45,46]. However, a key feature of MdDS that is present in both MT and NMT/SO populations is remission (or significant reduction) of symptoms during the re-exposure to passive motion [4], which does not occur in patients with PPPD. The data in this study revealed 6% of NMT/SO respondents attributed the onset of their symptoms to an injury or accident. Thus, it could be argued that these types of NMT/SO patients, specifically, could be categorized as a subset of PPPD, since the umbrella classification of PPPD also includes chronic dizziness that has developed following a head injury [47], or that these individuals may have VH, which is known to occur after mTBI [14].

#### Age at onset

When analyzing the two MdDS groups, the NMT/SO respondents were significantly younger on average at the age of MdDS onset compared with the MT respondents (38.3 and 44.1 years, respectively). The mean age of onset in the MT group was in accordance with other studies, i.e., within the 5th decade of life (40–50 years) [4,35,48]. At this age, many women experience perimenopause or the menopausal transition, which is associated with significant hormonal fluctuations [40,49,50]. These fluctuations, particularly regarding estrogen, have been hypothesized to play a role in MdDS pathophysiology and onset. Potentially, aberrant hormonal phases such as pre-menopausal and menopausal states are predisposing factors, rendering those who go on to develop MT MdDS more susceptible at the time of onset [3]. The mean age of onset in the NMT/SO group was below 40 years of age, supporting that the two subtypes of MdDS indeed may have different pathophysiological mechanisms [3,40]. In alignment with a previous study, premenopausal NMT/SO patients were more sensitive to the monthly hormonal fluctuations that affected their symptoms and displayed a greater incidence of menses irregularity when compared the MT group [3].

Sex hormones have been shown to be involved in certain ocular pathologies, with numerous gonadal hormone receptors located in various regions of the eye itself [51]. Fluctuating estrogen levels have been directly linked to changes in visual function due to altered refractive error, corneal thickness and retinal dysfunction [52,53]. Therefore, it could be suggested that the hormonal fluctuations, typical of menopausal transition, and the hormonal dysregulation commonly observed, could also be involved in contributing to visual dysfunction, which in turn makes these individuals more susceptible to developing MdDS, or leads to symptoms which are commonly associated with MdDS and VH. The number of studies on ocular mobility and ocular function changes with different hormonal stages are still limited, this aspect requires further exploration given the recent hypothesis between MdDS and hormonal fluctuations [41].

#### Associated symptoms

The most common associated symptoms in both subtypes were unsteadiness, brain fog, light sensitivity and anxiety, with more than 63% and 68% of the MT and NMT/SO participants reporting these symptoms, respectively. These symptoms are common, not only in MdDS, but are also reported by people with VH; hence a careful evaluation is critical, to ensure patients are treated appropriately for MdDS, VH or both. Unfortunately, these symptoms are vague and expressed by patients with other neurological and neurotologic disorders, anxiety and other vestibular disorders. As a result, patient history reviews must be comprehensive and inquire deeper into details such as visual symptoms in these cases. Interestingly, symptoms associated with vision disorders such as double vision, nausea and ‘shaking’ peripheral vision were more prevalent in the NMT/SO group (occurring in 34–42% of the group) compared with the MT group (10–27% of the group), further suggesting that NMT/SO patients may have an underlying visual disturbance or visual disorder because their symptoms are similar to patients with ocular misalignment rather than patients with MT MdDS [26,54]. Taking this into consideration, it may be beneficial for NMT/SO patients to undergo BVD evaluation and treatment, if appropriate.

### The general population group versus people with MdDS

The BVDQ was used as it provides an overall score that identifies the probability of VH within vestibular patients, and it is comprehensive in nature due to its considerations into various symptom domains (i.e., 1. Headache, 2. Head tilt, 3. Vestibular, 4. Anxiety, 5. Binocular vision, 6. Reading, and 7. Standard vision). In this study, all symptom domains were considered, except for reading and anxiety. The overall mean BVDQ score of the MdDS group was higher than the healthy controls (32 vs 12, respectively), with a score over 30 indicating a high probability of VH. Specifically, when considering the headache domain; around one fifth of the MdDS respondents indicated that they always ‘have a headache or facial pain’ and ‘pain while moving their eyes’, whereas the general population did not report ‘always’ experiencing either at all. The higher rate of headaches and migraine has been reported in the MdDS population previously, and migraine medications have been previously recommended, though have had little success [39,43,46,55]. It is important to note that certain visual disorders (such as glaucoma, refractive disorders, heterophorias, etc.) are also associated with frequent headaches and facial pain [56,57]. The higher rate of ‘pain while moving the eyes’ in the MdDS group was an interesting find, as this symptom is generally associated with ocular disorders such as refractive anomalies and phorias, inflammation of ocular structures (i.e., optic neuritis, ocular myositis, uveitis, etc. [58,59]). Regarding scores in the head tilt domain (given that when BVD is present, patients tend to tilt their head as compensatory mechanism [9]), almost 80% of the MdDS respondents indicated that they frequently/always ‘experience neck and shoulder discomfort’ and 55% frequently/always ‘find themselves with their head tilted to one side’. This suggests that there is a high probability of some sort of visual dysfunction in the MdDS clinical population which requires further investigation. In addition, the NMT/SO group had a higher proportion of neck and shoulder discomfort compared with the MT group, which further supports that NMT/SO patients may be even more affected by visual dysfunction. This data, despite being unconfirmed by a clinical ophthalmological examination, is extremely important, given that these patients might have an undiagnosed vertical misalignment that could be managed with the adoption of prisms lenses, as shown in Rosner *et al.* [9]. This higher likelihood of visual dysfunction in the NMT/SO population could also explain why this subtype of patients are less responsive to optokinetic (OKN) stimulation and report a higher degree of migraine complaints [3]. The vestibular domain results indicated that the MdDS group had a higher proportion of individuals ‘always’ experiencing various vestibular symptoms when compared with the general population. This was expected, however when comparing the MdDS subtypes, it was interesting to see that a greater proportion of NMT/SO subjects experienced dizziness/light headedness, or nausea while performing close-up activities (by twofold) and far-distance activities (by fourfold). It is known that oculomotor system deficits lead to a diminished visual accommodation reflex [60] and visual impairments can lead to sustained and systematic oculomotor load and affect balance mechanisms [61]. This further supports the presence of visual dysfunction in the NMT/SO population. In the binocular vision domain, no differences were reported between the subtypes, though, overall, the MdDS group reported higher levels of binocular dysfunction compared with the general population. This included issues with depth perception, double/overlapped/shadowed vision at far and near distances and photosensitivity. Though the photosensitivity could be attributed to sensory reweighting toward the visual system due to vestibular dysfunction, depth perception is considered exclusively vision-based [62] and not involving the vestibular system. In addition, the double/overlapped/shadowed vision at far and near distances again highlights the potential of visual dysfunction in the MdDS population. In the standard vision domain, no differences were reported between the subtypes, though, overall, the MdDS group reported higher levels of standard vision dysfunction compared with the general population. This included visual fatigue and blurred vision with close-up tasks and blinking to ‘clear up’ distant objects after working at a desk or working with closeup activities. Again, these symptoms and behaviors may be due to an over-worked visual system due to sensory reweighting, though it really highlights the potential of the presence of visual disorders, that could be addressed if identified.

#### Visual disorders & visual health

##### Visually induced dizziness

Of the previously diagnosed visual disorders (i.e., patients had a formal diagnosis), there was a higher rate of VID in the MdDS group, where MdDS respondents were 22-times more likely to have VID than the general population, with no differences between the two subtypes. This, however, does not consider individuals who were not aware of having VID, so does not accurately represent the true proportion in each group. However, the VVAS and SVQ questionnaires measure the patient's experience of vertigo and dizziness in visually challenging environments (e.g., high contrast wall or floor coverings, bright, flashing lights, etc.). In both questionnaires, the MdDS group demonstrated higher scores compared with the general population group, with no differences between the MdDS subtypes. This is in line with previous studies, where an oversensitivity to visual stimuli has been reported in MdDS patients [2,4,5,35], and can lend further support for the role of vision pathology in this clinical population. The presence of VID may be due to an undiagnosed VH or could be the result of a compensatory adaptation to MdDS, involving sensory reweighting [63]. The etiology of visual motion discomfort and sensitivity in MdDS patients remains unclear. It has been hypothesized that it is the result of a non-compensatory mechanism, i.e., patients become more sensitive to visual motion due to a maladapted VOR or to an over reliability on the visual system to orient and maintain balance [63,64]. Further examination is essential to confirm whether they are associated symptoms of MdDS, or comorbidities, and understanding when VID develops in MdDS patients would be crucial. It is also known that patients suffering from VH are more likely to experience visually induced motion sickness [65]. Therefore, a visual examination of MdDS patients is crucial to understanding the potential etiology of their discomfort.

##### Binocular visual dysfunction

Binocular visual dysfunction was another previously diagnosed visual disorder that had a higher frequency in the MdDS group, with no differences between the two subtypes. Again, this does not consider individuals who were not aware of having BVD. However, the BVDQ, a validated VH symptom assessment instrument, was used to evaluate potential VH symptom frequency. Higher BVDQ scores were recorded in the MdDS group compared with the general population. The presence of VH in MdDS may be highly relevant to the future management of these patients. Between the two subtypes, the NMT/SO group had a significantly higher average BVDQ score compared with MT respondents, suggesting that the NMT/SO may have a greater degree of VH or BVD, which predisposes them to nausea, ‘shaking’ peripheral vision and double vision. These findings suggest that patients displaying typical MdDS symptoms should be closely evaluated for VH or BVD too, to rule out visual misalignments.

Visual misalignments have been shown to be a limiting factor in vestibular rehabilitation (VR), as described by Pavlou *et al.* [26] on patients suffering from visual vertigo (also known as VID). In their study, after an orthoptic assessment, patients were categorized as either having or not having a binocular vision abnormality. All patients received VR and it was found that the visual vertigo patients without binocular vision abnormalities responded better to VR, compared with those who had binocular vision abnormalities [26]. This was proposed to be based on differences in fixation during the treatment, which was OKN exposure. When considering the OKN treatment currently available for MdDS patients [1,66], despite the promising results, some patients do not respond to the therapy [64]. The success rate of the OKN treatment is around 70% in MT patients [1,64] (which reduces to 50% success ~1 year follow-up [66]) and 30% in NMT/SO [64]. The lower success rate reported in the NMT/SO group could be due to a higher rate of BVD. A possible explanation may be that some patients may have had a pre-existing, undiagnosed VH, which could lead to a non-symmetrical OKN reflex and an abnormal sensitivity to higher speed visual motion. This is relevant to MdDS patients because the OKN system plays a key role in gaze stabilization, visual adjustments in relation to postural control, volitional smooth eye movements and visually guided motor learning [67]. It is plausible that the coexistence of VH may be a key factor limiting the ability of these patients to suppress postural sway responses to visual stimuli and why some patients respond better to rehabilitation than others. It has also been shown that the two MdDS subtypes respond differently to traditional vestibular therapy with the MT subtype finding it somewhat helpful, and not helpful at all in the NMT/SO subtype [38]. Our introductory findings recommend the need to thoroughly examine this aspect in MdDS patients.

##### Visual correction & eye operations

Prescription glasses and contact lens usage and rate of eye operations was similar between the general population and the MdDS group. However, when comparing the two subtypes, the MT group had a higher rate of eye operations than the NMT/SO group, though this was not significant. We propose that perhaps the higher number of reports of visual disturbances in the NMT/SO group (i.e., ‘shaking’ peripheral vision, double vision and high BVDQ scores), could be because these patients did not have their pre-existing visual deficits adequately diagnosed or corrected, as they reported fewer eye surgeries compared with the MT group. Our early findings highlight the importance of examining the visual and oculomotor systems history in MdDS patients. Eye examinations, including binocular vision testing, will be essential to understanding if MdDS patients have an underlying vertical misalignment or vertical phoria that may play a role in their symptom experiences.

#### History of mild traumatic brain injury

mTBI (concussion) is known to cause visual misalignments, refractory dizziness, vertigo, nausea, dizziness, brain fog, unsteady gait and postural instability, VOR disruptions and oculo-motor abnormalities [12,68–74]. These symptoms are also often described by MdDS subjects. No difference was reported in the occurrence of mTBI in the general population group and MdDS group, 9.1% and 11.7%, respectively. There was, however, a statistically significant difference between the two MdDS subtypes: one quarter of the NMT/SO group sustained mTBI (concussion), which was three-times the number of the MT patient group (as a proportion). This finding raises critical questions about the underlying pathophysiology of patients with NMT/SO onset. mTBI is associated with neurocognitive impairment, visual and vestibular disturbances and postural instability as mentioned previously [70,71,75]. In the past few years, Staab and colleagues have categorized mTBI post-concussion dizziness under the umbrella of PPPD [45]. This poses the question if NMT/SO MdDS patients that have a history of mTBI should also fall into the PPPD umbrella category. Further strategic research of patients with mTBI is required to understand the underlying cause(s) of their chronic dizziness. Regardless, NMT/SO patients require a comprehensive neurologic, neuro-otologic and neuro-ophthalmic examination. It is plausible that chronic dizziness is multi-factorial and due to different etiologies to what was thought some decades ago. This finding underscores the importance of detailed patient histories, a multi-disciplinary team approach and the relevance of differential diagnoses.

### Study strengths & limitations

This study was quasi-explorative because we are seeking data to justify further investigations. The major strength of this study was the unprecedented data collection of visual disorders, visual health and associated comorbidities, such as headaches, medical procedures and mTBI (concussion), in the MdDS population. An examination of patient history, especially of the NMT/SO MdDS patients, has provided us with a larger and more robust body of information. This may provide us with a greater insight into the onset and development of MdDS symptoms, potentially overlooked in previous comparative studies [3,4]. Our results indicate the questions pertinent to mTBI (concussion) were noteworthy; hence we recommend MdDS patients be screened for post-concussion dizziness as well as VH. One of the limitations of our study was the recruitment of participants, as we were limited to those active on social media and those who visited web pages related to our studies. This may have introduced some sampling bias, particularly if a patient's condition was so severe that they could not engage in computer use (due to cybersickness and heightened VID symptoms) or missing older patients with less computer use or skills. Also, the diagnosis and treatment of the MdDS population is widely scattered across different medical specialties, as well as significantly undiagnosed by the medical system; there is little opportunity to obtain a truly random sample. However, we believe this limitation is relevant for all online surveys on MdDS patients, visual and vestibular conditions. The lack of access to patients' full medical records and being unable to physically examine patients were additional limitations. We understand these are common problems associated with online studies recruiting large groups of international participants, and access to full medical records are either not available or overlooked. In the future, we hope a secure cloud platform becomes available for researchers to use which will enable international telemedicine studies. We propose that medical records and objective screening are essential for accurate differential diagnoses. Like previous surveys, some questions requested information about events which occurred many years before, and, as such, there are no specific strategies for controlling recall bias.

### Future research

Further research is necessary for several reasons: first, to validate and objectify the clinical symptoms in patients with MdDS, thereby differentiating the two MdDS subtypes. Second, to differentiate MdDS from other vestibular and visual disorders with accurate biomarkers. Given the subjective nature of these conditions, neuroimaging studies are essential to identify potential biomarkers to improve the diagnosis of these patients. Comparative studies of the two subtypes of MdDS and other patient groups such as PPPD, VH, mTBI post-concussion dizziness, clinically depressed patients with and without dizziness and vestibular migraine (VM) are needed. A comprehensive general and binocular vision examination of all MdDS patients is critical to advance our understanding of these patients, as to whether they suffer from an underlying eye condition or visual disorders that potentially predispose them to develop MdDS. In the future, the approach should be broad and not only compare MdDS subtypes but also other disorders that share similar symptoms. Comparing different groups that share similar clinical features may shed light on the specific neural pathways involved and enable the identification of specific biomarkers, which may provide targets for future treatment interventions. Overall, we suggest developing further studies that will serve as a foundation for the development of differential diagnostic guidelines.

## Conclusion

This was the first study to investigate visual disorders, visual dysfunction, mTBI and general visual health data in the MdDS population. Our hypotheses were confirmed that a higher rate of visual disorders and visual dysfunctions would be observed in the MdDS patients compared with the general population, and that exploring headache experience and medical histories (mild head injuries and eye operations) would reveal distinguishing features between the MdDS patients and the general population, and between the two MdDS subtypes. The major findings of this study were twofold: first, the prevalence of vision-related symptoms, such as frequent headaches and eye-movement, neck and shoulder pain, depth perception issues and visual disturbances, and the prevalence of visual disorders, namely VH and VID, were significantly higher in the MdDS population compared with the general population group. Moreover, the MdDS patients displayed symptoms that have been reported in people who suffer VH. Additionally, the study highlighted unique differences between the two MdDS subtypes, specifically the NMT/SO group had a lower age of onset on average, a significantly higher proportion of males, a higher prevalence of visual disturbances (double vision and ‘shaking’ peripheral vision) and vision-related symptoms (neck and shoulder discomfort, dizziness/light headedness, or nausea while performing close-up and far-distance activities), lower number of eye-related surgeries or interventions, higher BVDQ scores on average and a greater number of mTBI (concussions) compared with the MT group. This suggests that visual disorders coexist with MdDS and might be more prevalent in the NMT/SO group. These differences underscore the crucial need for further research of both MdDS subtypes and their differences. It is imperative to establish a clear classification for NMT/SO patients that will assist healthcare practitioners and patients. Is NMT/SO a subtype of MdDS or a condition with a different etiology and similar symptoms to MdDS? In conclusion, we have demonstrated unequivocal evidence that it is essential that clinicians assess MdDS patients for VH, as its presence will influence the treatment of MdDS and how this aspect should be thoroughly evaluated. Patient evaluation, treatment and management can also be improved by providing a targeted and strategic approach for patient management, hence further research is imperative in this field. Despite being based on questionnaires, the findings presented emphasize the importance of a comprehensive patient history for all MdDS patients, inquiring about mTBI (concussion) in the NMT/SO group and collecting a detailed anamnesis. We recommend routine screening of VH and other potential ocular-motor dysfunctions to provide patients with a customized treatment and management program.

This study has provided us with valuable information to advance our understanding of this clinical population, and appropriate diagnostic assessments, treatments and management strategies.

## Future perspective

In the next 5–10 years, we would expect comparative studies (with clear biomarkers) that objectively validate the clinical differences in patients with MT and NMT/SO MdDS to have been conducted. In addition, we would also expect comparisons to be made in respect to other central vestibular disorders (e.g., PPPD, VM) and mTBI. We would hope that neuroimaging becomes a part of the standard-of-care to identify potential biomarkers, thereby facilitating a prompt diagnosis of these patients. We recommend longitudinal studies be conducted, incorporating multi-comparisons of different treatments ranging from pharmacological (e.g., migraine medications, antidepressant) to non-pharmacological treatments (including OKN rehabilitation, eye disorder management through prism glasses and neuromodulation). This would be of significant scientific value and clinical benefit. Comparing different groups with similar clinical features, to determine the specific neural pathways involved, would enable the potential identification of specific biomarkers to provide targets for future treatment interventions. We propose future research studies will provide the foundation for the development of differential diagnosis, treatment and management guidelines to advance our understanding of MdDS and offer these patients an improved quality of life.

**Summary points**

- A higher frequency of vision-related symptoms and disorders was present in the MdDS population compared with the general population.
- Our findings suggest visual disorders, such as VH, may coexist with MdDS, in particular, in the NMT/SO subtype.
- NMT/SO patients had a higher frequency of visual disturbances and vision-related symptoms than the MT patients.
- A history of mTBI was more frequent in the NMT/SO patients than the MT patients.
- Addressing co-existing visual disorders will lead to better treatment outcomes for both MdDS subtypes.
